# Clinical Risk Scoring Models for Prediction of Acute Kidney Injury after Living Donor Liver Transplantation: A Retrospective Observational Study

**DOI:** 10.1371/journal.pone.0136230

**Published:** 2015-08-24

**Authors:** Mi Hye Park, Haeng Seon Shim, Won Ho Kim, Hyo-Jin Kim, Dong Joon Kim, Seong-Ho Lee, Chung Su Kim, Mi Sook Gwak, Gaab Soo Kim

**Affiliations:** 1 Department of Anesthesiology and Pain Medicine, Samsung Medical Center, Sungkyunkwan University School of Medicine, Seoul, Republic of Korea; 2 Department of Anesthesiology and Pain Medicine, Samsung Changwon Hospital, Sungkyunkwan University School of Medicine, Changwon, Republic of Korea; University of Toledo, UNITED STATES

## Abstract

Acute kidney injury (AKI) is a frequent complication of liver transplantation and is associated with increased mortality. We identified the incidence and modifiable risk factors for AKI after living-donor liver transplantation (LDLT) and constructed risk scoring models for AKI prediction. We retrospectively reviewed 538 cases of LDLT. Multivariate logistic regression analysis was used to evaluate risk factors for the prediction of AKI as defined by the RIFLE criteria (RIFLE = *r*isk, *i*njury, *f*ailure, *l*oss, *e*nd stage). Three risk scoring models were developed in the retrospective cohort by including all variables that were significant in univariate analysis, or variables that were significant in multivariate analysis by backward or forward stepwise variable selection. The risk models were validated by way of cross-validation. The incidence of AKI was 27.3% (147/538) and 6.3% (34/538) required postoperative renal replacement therapy. Independent risk factors for AKI by multivariate analysis of forward stepwise variable selection included: body-mass index >27.5 kg/m^2^ [odds ratio (OR) 2.46, 95% confidence interval (CI) 1.32–4.55], serum albumin <3.5 mg/dl (OR 1.76, 95%CI 1.05–2.94), MELD (model for end-stage liver disease) score >20 (OR 2.01, 95%CI 1.17–3.44), operation time >600 min (OR 1.81, 95%CI 1.07–3.06), warm ischemic time >40 min (OR 2.61, 95%CI 1.55–4.38), postreperfusion syndrome (OR 2.96, 95%CI 1.55–4.38), mean blood glucose during the day of surgery >150 mg/dl (OR 1.66, 95%CI 1.01–2.70), cryoprecipitate > 6 units (OR 4.96, 95%CI 2.84–8.64), blood loss/body weight >60 ml/kg (OR 4.05, 95%CI 2.28–7.21), and calcineurin inhibitor use without combined mycophenolate mofetil (OR 1.87, 95%CI 1.14–3.06). Our risk models performed better than did a previously reported score by Utsumi et al. in our study cohort. Doses of calcineurin inhibitor should be reduced by combined use of mycophenolate mofetil to decrease postoperative AKI. Prospective randomized trials are required to address whether artificial modification of hypoalbuminemia, hyperglycemia and postreperfusion syndrome would decrease postoperative AKI in LDLT.

## Introduction

Acute kidney injury (AKI) has been reported to be a frequent complication after orthotopic liver transplantation (LT) which is associated with poor graft survival and increased mortality [1–6]. The incidence of AKI after liver transplantation ranges from 20 to 64% [1–3, 7–15], and postoperative AKI is associated with the development of chronic kidney disease [10, 16].

Currently, there is no effective therapy or preventive strategy available for AKI after LT [1], although several promising strategies were investigated [17–19]. Therefore, identifying modifiable risk factors and preventing postoperative AKI or early intervention is essential to improve outcomes [20]. Although a number of studies have evaluated AKI after LT, the incidence and clinical risk factors are not entirely clear and evidences regarding modifiable risk factors are still lacking. This ambiguity may be explained by the variable definitions used for AKI and different clinical settings used in previous studies [1–4, 7–9]. Furthermore, most previously-reported risk factors including longer anhepatic phase [12], intraoperative blood loss [1, 13], and large transfusion amount [3, 8, 15], model for end-stage liver disease (MELD) score [2, 7, 8, 12, 21] are not modifiable.

In addition, most previous studies involved deceased donor liver transplantation and, to our knowledge, only a few groups studied the risk factors of AKI after living donor LT (LDLT) [1, 8, 13, 14]. Utsumi et al.[1] suggested the usefulness of RIFLE criteria (RIFLE = risk, injury, failure, loss, end stage) in LDLT and highlighted LDLT-specific risk factor, graft to recipient body weight ratio (GRWR). Limitations of these studies [1, 8, 13, 14] are they were performed on a relatively small number of patients over a long time period and hemodynamic or metabolic risk factors including postreperfusion syndrome [22], vasopressor infusion during operation [15], preoperative hyperuricemia [23] and intraoperative hyperglycemia [24] have not been evaluated.

The purpose of this study was to determine the potentially modifiable risk factors, including hemodynamic and metabolic variables, that can identify patients at high-risk for AKI after LDLT. The second aim was to develop and validate specific clinical risk score models that accurately predict AKI after LDLT.

## Material and Methods

### Design and Patients

**Fig 1:**
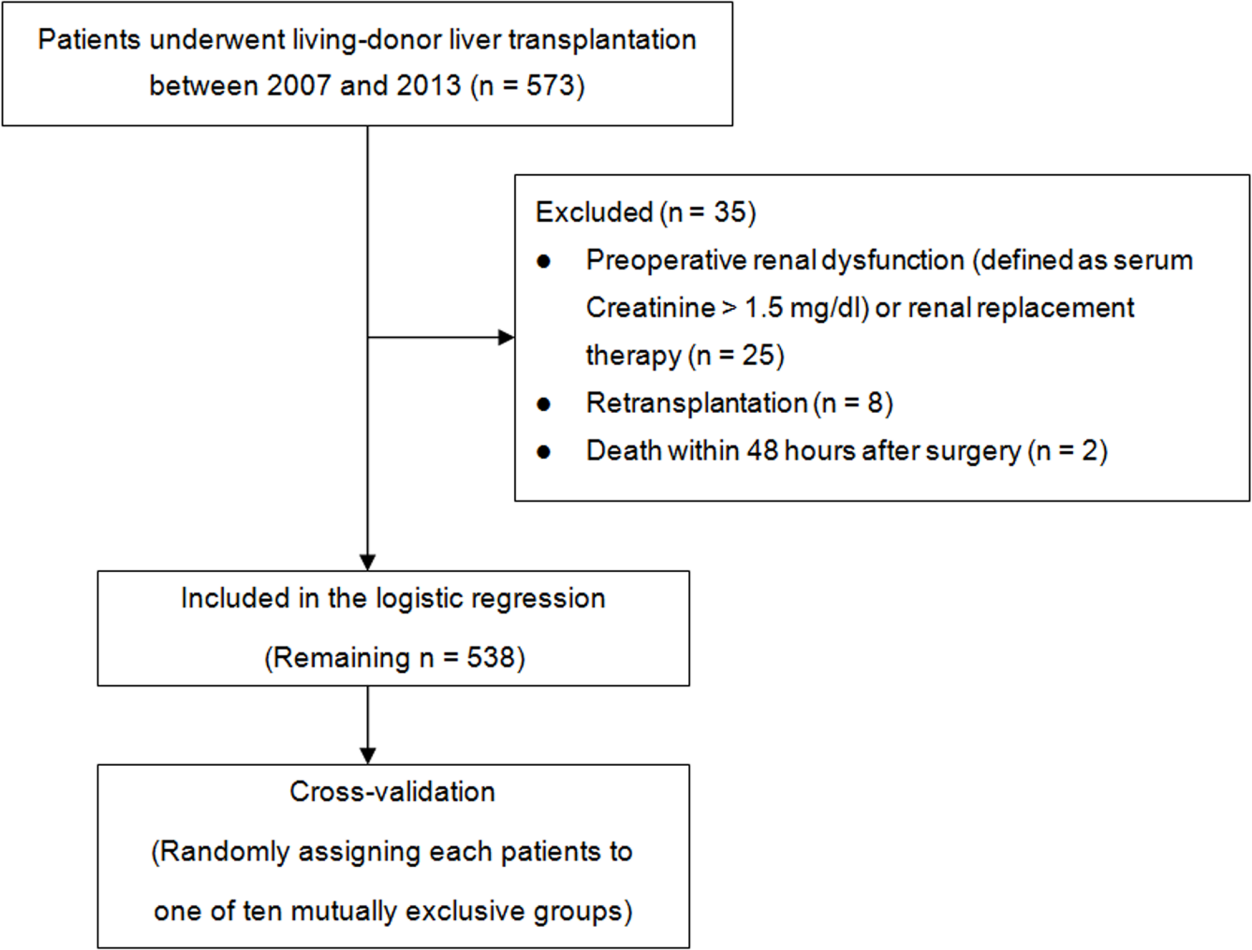
Flow diagram outlining the inclusion and exclusion criteria and study design.

This retrospective observational study was approved by the institutional review board of our institution (Samsung Medical Center IRB: 2013-12-080-001). We retrospectively reviewed the electronic medical records of 573 consecutive adult patients who underwent LDLT at our institution between 2007 and 2013 (Fig 1). This study was registered at http://www.clinicaltrials.gov (NCT02080065). The need for informed consent was waived given the study’s retrospective design. Patients with preoperative renal dysfunction (defined as serum creatinine >1.5 mg/dl) or renal replacement therapy (RRT) were excluded (n = 25). Patients who underwent retransplantation (n = 8) or died within 48 hours postoperatively (n = 2) were excluded. The remaining 538 patients were analyzed.

### Anesthesia, Surgical Technique and Immunosuppression

The followings are anesthesia and surgical technique used during the study period. Continuous inhalation anesthesia with desflurane and continuous remifentanil infusion were administered [25, 26]. Donor grafts were prepared with histidine–tryptophan–ketoglutarate (HTK) solution. The piggyback technique was used to anastomose the graft and donor vessels. Neither a venovenous bypass nor a temporary portocaval shunt was used. End-to-end anastomosis of the hepatic artery and duct-to-duct anastomosis of the bile duct were carried out in succession. All liver recipients were transported to an intensive care unit for postoperative care. During surgery, immunosuppression was induced with 500 mg methylprednisolone (Solumedrol, Pfizer, Ballerup, Denmark) and 20 mg basiliximab I.V. (Simulect, Novartis Pharma B.V., Arnhem, Netherlands). During the postoperative period, immunosuppression was induced with calcineurin inhibitors (CNI) of either tacrolimus or cyclosporine. The blood level of tacrolimus was adjusted to maintain a target plasma concentration of 10–15 ng/ml during the first month; this level was reduced to 5–10 ng/ml thereafter. Target blood level of cyclosporine was 150–400 ng/ml during 90 days and 100–300 ng/ml thereafter. The CNI dosage was adjusted according to the whole blood trough level measured 12 h after the initial postoperative administration. During the retrospective cohort study period, the CNI blood level measurement protocol was not changed. Mycophenolate mofetil (MMF) was given additionally to reduce the CNI dosage, lower the CNI trough level and reduce potential CNI toxicity for selected patients with high CNI trough level.

### Data Collection

Based on previous literature, data related to demographic or perioperative variables known to be related to postoperative renal dysfunction were collected [1–3, 7, 8, 12, 13, 15, 22–24]. Preoperatively, the Model for End-stage Liver Disease (MELD) score, the Child-Turcotte-Pugh (CTP) score and the child classification were determined for all recipients [27]. Postreperfusion syndrome was determined when the mean arterial pressure was 30% lower than the previous value at the end of anhepatic stage and lasted for at least 1 minute during the 5 minutes after reperfusion by reviewing the electronic anesthesia medical record [22, 28]. Norepinephrine infusion was recorded when norepinephrine was infused more than one hour during the surgery. Norepinephrine was started to be infused when measured systemic vascular resistance was less than 600 dyne sec/cm^5^ and mean blood pressure less than 70 mmHg despite infusion of dopamine during the study period. Mean blood glucose levels were calculated by averaging all the measurements during the day of surgery. Rapid insulin was infused at a rate of 5 units/hour when the blood glucose level was greater than 150 mg/dl and 10 units/hour when it was greater than 200 mg/dl. Rapid insulin 10 units were administered when serum potassium was greater than 4.5 mEq/L before reperfusion. These protocols were not changed during the study period.

The primary outcome variable was postoperative AKI. AKI was defined according to the RIFLE criteria (RIFLE = *r*isk, *i*njury, *f*ailure, *l*oss, *e*nd stage), which have been validated in patients undergoing liver transplantation [1, 11, 12, 29]. We determined postoperative AKI according to RIFLE criteria based on the maximal change in sCr level [30] and eGFR [31] during one month of postoperative follow-up. A Urine output criterion was not used. All patients who met the RIFLE criteria for Risk, Injury, and Failure were classified as having AKI. Classes L and E were not used and RRT was defined as a new dialysis requirement. Postoperative clinical outcome variables included postoperative RRT, length of ICU stay, length of hospital stay, and mortality rates during hospital stay and within one year of surgery.

### Statistical Analysis

SPSS software version 21.0 (IBM Corp., Armonk, NY, USA) was used to analyze the data. For all analyses, *p*<0.05 was considered statistically significant. Sample size was validated under the assumption that the result of a risk model should have more than 10 outcome events per independent risk factor for accuracy [32]. Using this information, we estimated that 333 patients or more were needed in the cohort to allow ten or fewer predictor variables in a multiple logistic regression model (estimated 30% rate of postoperative AKI).

Categorical variables were reported as absolute number (*n*) and relative frequency (%), and continuous variables were reported as median (interquartile range). The Kolmogorov-Smirnov test was used to determine the normality of the data. Either Fisher’s exact test or χ^2^ test was used to compare the categorical variables against the RIFLE criteria. Comparisons of continuous variables between AKI and non-AKI patients were performed with Mann-Whitney *U* tests. Logistic regression models were used to identify univariate and multivariate predictors for AKI. Univariate logistic regression analysis was used to identify possible risk factors for AKI. Next, the multivariate model only included variables that were found to be significant AKI risk factors on univariate analysis (*p*<0.05). Three risk scoring models were developed in the retrospective cohort by including all variables that were significant in univariate analysis (Model 1), or variables that were significant in stepwise multivariate analysis by backward stepwise variable selection (Model 2), or forward stepwise variable selection (Model 3) with a significance criterion of *p*<0.05. A cut-off point with the maximal sum of sensitivity and specificity was determined on the receiver operating characteristic (ROC) curve. This cut-off point or a point used in previous study was used to categorize the continuous variables in developing the final logistic regression model. Missing data were present in less than 2% of records. Missing values for continuous variables were assigned the gender-specific median values and categorical values were assigned the most frequent gender-specific values.

Risk scoring models used the odds ratio of variables in the multivariate analysis. Our risk models were validated by way of cross-validation [33, 34]. Our risk scoring models were applied to randomly generated validation samples by selecting mutually exclusive ten subgroups of our cohort to assess the accuracy of the score’s AKI prediction. To measure and compare the predictive accuracy of the developed risk scores, we generated the ROC curves and compared their C-statistics [35]. Calibration of the risk score was assessed using Hosmer-Lemeshow goodness-of-fit statistics. We also compared the performance of our scoring model, as measured by AUC, to a previous predictive index by Utsumi et al. in our retrospective cohort [7]. Delong’s methods were used to compare the AUC among AKI risk models in this study with those of the previously reported index [36].

## Results

During the first postoperative month, AKI (as determined by RIFLE criteria with 18.2% Risk, 7.8% Injury and 1.3% Failure) occurred in 147 patients (27.3%) in the retrospective cohort. Thirty-four patients (6.3%) required RRT. Patient demographics and perioperative variables according to the diagnosis of AKI are presented in Table 1. Postoperative ICU stay was significantly longer and in-hospital mortality was significantly higher in patients with AKI.

**Table 1:** Patients characteristics and perioperative parameters by RIFLE classification.

		RIFLE criteria				
Characteristics	No AKI	Risk	Injury	Failure	p-value^a^	p-value^b^
**Patient population, n**	391 (72.7)	98 (18.2)	42 (7.8)	7 (1.3)		
**Demographic data**						
** Age, years**	54 [49–58]	53 [46–57]	55 [49–58]	60 [49–62]	0.548	0.197
** Female, n**	76 (19.4)	23 (23.5)	7 (16.7)	2 (28.6)	0.547	0.638
** Body-mass index, kg/m** ^**2**^	23.3 [21.2–25.8]	23.8 [21.5–26.1]	24.6 [21.5–29.3]	26.0 [23.4–30.1]	0.017	0.032
**Background medical status**						
** Hypertension, n**	39 (10.0)	12 (12.2)	2 (4.8)	3 (42.9)	0.597	0.044
** Diabetes mellitus, n**	63 (16.2)	20 (20.4)	14 (33.3)	2 (28.6)	0.026	0.035
** Smoking history, n**	143 (36.6)	45 (45.9)	12 (28.6)	4 (57.1)	0.294	0.127
** Smoking history, pack-years**	15 [10–30]	13 [6–20]	11 [3–19]	-	0.134	0.390
** Alcoholic liver cirrhosis, n**	31 (7.9)	18 (18.4)	5 (11.9)	3 (42.9)	0.001	0.001
** HBV hepatitis, n**	263 (67.3)	63 (64.3)	20 (47.6)	2 (28.6)	0.041	0.015
** HCV hepatitis, n**	30 (7.7)	4 (4.1)	5 (11.9)	-	0.537	0.346
** Hepatocellular carcinoma, n**	259 (66.2)	48 (49.0)	17 (40.5)	1 (14.3)	<0.001	<0.001
** Cholestatic disease, n**	12 (3.1)	2 (2.0)	1 (2.4)	-	0.770	0.921
** Serum albumin level, mg/dl**	3.4 [3.0–3.9]	3.3 [2.8–3.6]	2.0 [2.7–3.4]	2.9 [2.7–3.2]	<0.001	<0.001
** Uric acid, mg/dl**	5.1 [3.4–6.7]	5.1 [3.6–7.2]	5.7 [4.4–7.1]	6.9 [4.4–7.5]	0.179	0.293
** MELD score**	12 [8–17]	14 [12–30]	18 [15–33]	24 [19–36]	<0.001	<0.001
** CTP score**	7 [5–10]	8 [7–11]	10 [8–11]	10 [10–11]	<0.001	<0.001
** Child class, n, (A/ B/ C)**	166/122/99	21/35/42	5/14/23	0/1/6	<0.001	<0.001
**Baseline renal function**						
** Preoperative serum creatinine, mg/dL**	0.77 [0.64–0.89]	0.74 [0.62–0.94]	0.91 [0.68–1.19]	0.78 [0.39–1.21]	0.365	0.026
** Preoperative estimated GFR, ml/min/1.73 m** ^**2**^	101 [86–120]	106 [77–127]	82 [62–111]	101 [75–125]	0.214	0.037
**Donor/ graft factors**						
** Age, years**	31 [24–41]	31 [25–42]	34 [25–45]	41 [32–49]	0.145	0.171
** Estimated GRWR**	1.10 [0.91–1.29]	1.02 [0.90–1.40]	0.86 [0.75–1.00]	0.80 [0.74–1.09]	0.001	<0.001
** Estimated GRWR < 0.8, n**	34 (8.2)	11 (14.9)	11 (26.8)	3 (42.9)	<0.001	<0.001
**Operation and anesthesia details**						
** Operation time, min**	531 [480–597]	526 [480–611]	594 [532–672]	571 [562–620]	0.018	0.002
** Cold ischemic time, min**	80 [66–98]	88 [68–106]	82 [71–164]	108 [82–445]	0.006	0.009
** Warm ischemic time, min**	30 [23–39]	33 [25–45]	38 [33–45]	45 [27–46]	<0.001	<0.001
** Colloid, ml**	1500 [1000–1500]	1500 [1000–1500]	1500 [1000–1500]	1500 [1000–1500]	0.019	0.109
**Postreperfusion syndrome, n**	45 (11.5)	25 (25.5)	10 (23.8)	2 (28.6)	<0.001	0.001
** Continuous infusion of norepinephrine, n**	242 (61.9)	53 (54.1)	28 (66.7)	4 (57.1)	0.389	0.429
** Continuous infusion of norepinephrine >0.1 μg/kg/min**	71 (18.2)	26 (26.5)	13 (31.0)	3 (42.9)	0.008	0.032
** Intraoperative mean blood glucose, mg/dl**	136 [109–165]	142 [120–159]	167 [157–178]	158 [156–189]	<0.001	<0.001
** Mean blood glucose > 180 mg/dl during the day of surgery, n**	65 (16.6)	18 (18.4)	5 (11.9)	3 (42.9)	0.769	0.238
** Mean blood glucose > 150 mg/dl during the day of surgery**	146 (37.3)	39 (39.8)	33 (78.6)	6 (85.7)	0.001	<0.001
** Intraoperative insulin administration, units**	10 [0–10]	10 [0–10]	10 [5–10]	10 [5–15]	0.181	0.298
** pRBC transfusion, units**	1 [0–2]	4 [2–4]	4 [0–4]	4 [1–24]	<0.001	<0.001
** FFP transfusion, units**	0 [0–2]	4 [2–5]	4 [0–5]	4 [0–10]	<0.001	<0.001
** Platelet transfusion, units**	0 [0–8]	6 [6–6]	6 [0–6]	6 [6–8]	<0.001	<0.001
** Cryoprecipitate transfusion, units**	0 [0–6]	6 [3–6]	6 [0–6]	6 [6–8]	<0.001	<0.001
** Blood loss per body weight, ml/kg**	34 [21–47]	77 [37–113]	55 [29–98]	86 [39–180]	<0.001	<0.001
**Postoperative factors**						
** Initial induction of CNI with tacrolimus, n**	338 (88.5)	95 (96.9)	38 (90.5)	5 (71.4)	0.064	0.015
** Initial induction of CNI with cyclosporine, n**	44 (11.5)	3 (3.1)	4 (9.5)	2 (3.8)	0.064	0.015
** Tacrolimus average trough level, ng/ml**	10.1 [9.7–10.4]	10.0 [9.7–10.3]	10.3 [10.2–10.8]	10.8 [10.8–11.4]	0.194	<0.001
** Cyclosporine average trough level, ng/ml**	173.2 [163.2–180]	154 [154–154]	176 [169–182]	-	0.718	0.088
** Mycophenolate mofetil use with CNI dose reduction**	213 (54.5)	39 (39.8)	12 (28.6)	3 (42.9)	<0.001	0.001
** Overexposure to CNI, n**	28 (7.3)	8 (8.2)	3 (7.3)	1 (14.3)	0.714	0.747
**Outcome**						
** Renal replacement therapy, n**	5 (1.3)	10 (10.2)	13 (31.0)	6 (85.7)	<0.001	<0.001
** Postoperative ICU stay, days**	6 [6–7]	6 [6–9]	7 [6–18]	11 [10–85]	<0.001	<0.001
** Length of Hospital stay, days**	29 [23–42]	31 [21–43]	33 [26–47]	46 [16–108]	0.633	0.403
** In-hospital mortality, n**	23 (5.9)	11 (11.2)	13 (31.0)	2 (28.6)	<0.001	<0.001
** One-year mortality, n**	53 (13.7)	11 (11.2)	16 (38.1)	2 (28.6)	0.084	<0.001

The values are expressed as the median [interquartile range] or number (%). AKI = acute kidney injury; HBV = hepatitis B virus; HCV = hepatitis C virus; MELD score = model for end-stage liver disease score; CTP score = Child-Turcotte-Pugh score; GRWR = graft to recipient body weight ratio; GFR = glomerular filtration rate; pRBC = packed red blood cell; FFP = fresh frozen plasma; CNI = calcineurin inhibitor.

^a^ comparing patients without AKI to all patients with AKI. p-values^a^ are the results of unpaired *t*-test or Mann-Whitney *U* test for continuous variables, and χ^2^ test or Fisher’s exact test for categorical variables.

^b^ comparing patients within four groups (no AKI, risk, injury, and failure). p-values^b^ are the results of One-way analysis of variance or Kruskal-Wallis test for continuous variables, and χ^2^ test or Fisher’s exact test for categorical variables.

**Fig 2:**
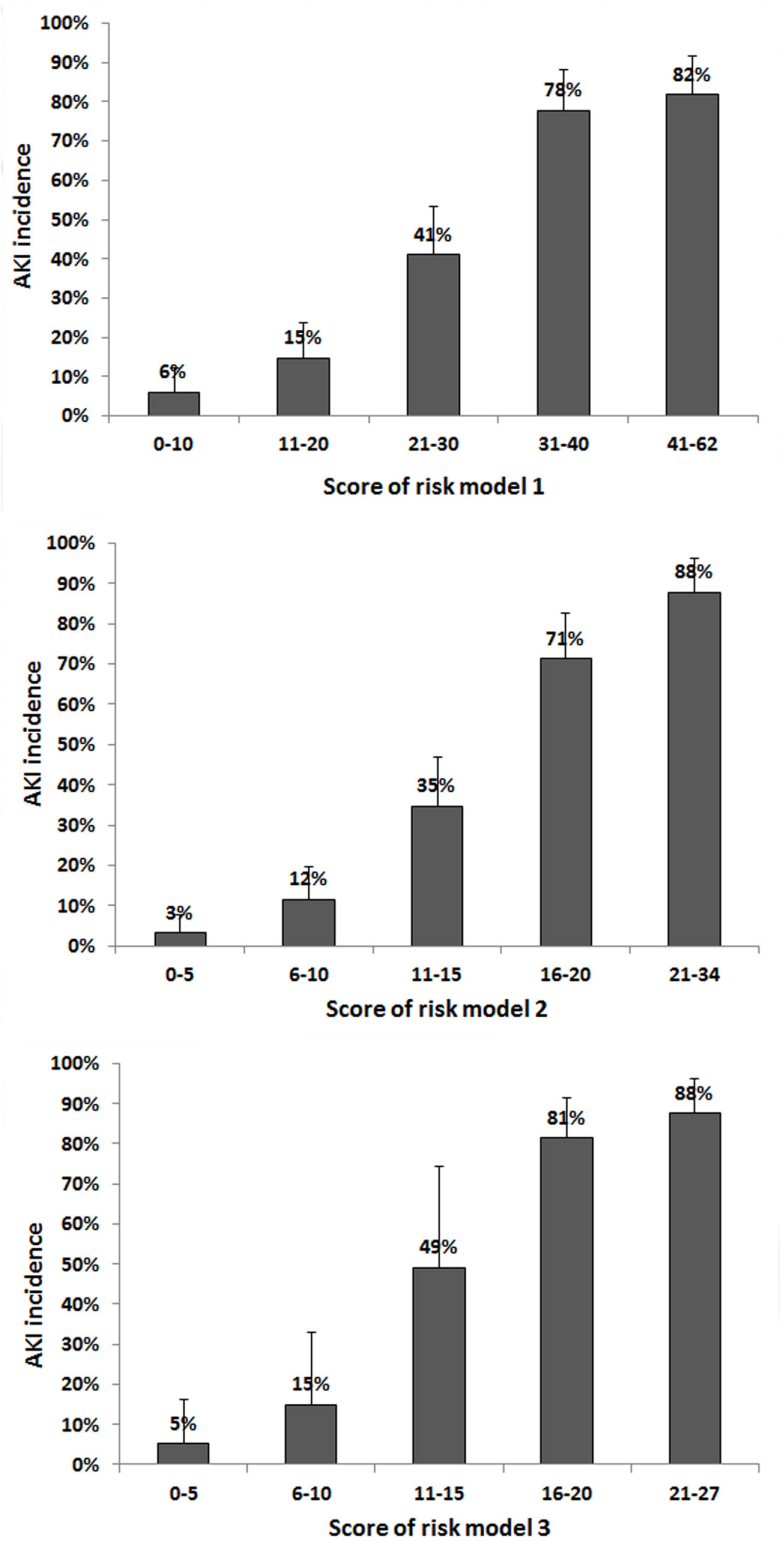
Proportion of patients with postoperative AKI within strata intervals of the three AKI risk scores. The numbers on the bar denote the percentage of patients with postoperative AKI in each score interval.

The results of univariate and multivariate analysis of the AKI risk factors within all RIFLE classes are shown in Table 2. Clinical risk-scoring models were developed by using odds ratio of predictors that were significant in univariate analysis (risk model 1), variables that were significant in stepwise multivariate analysis by backward stepwise variable selection (Model 2), or forward stepwise variable selection (Model 3). Independent risk factors for AKI of model 3 included: body-mass index >27.5 kg/m^2^ [odds ratio (OR) 2.46], serum albumin <3.5 mg/dl (OR 1.76), MELD score >20 (OR 2.01) operation time >600 min (OR 1.81), warm ischemic time >40 min (OR 2.61), postreperfusion syndrome (OR 2.96), mean blood glucose during the day of surgery >150 mg/dl (OR 1.66), cryoprecipitate > 6 units, blood loss/body weight >60 ml/kg (OR 4.05), and calcineurin inhibitor use without combined mycophenolate mofetil (OR 1.87). The incidences of AKI at each risk score interval of all three risk scores were shown (Fig 2). Higher risk score had a graded association with a higher incidence of AKI.

**Table 2:** Logistic regression analyses of categorized risk factors for acute kidney injury within all RIFLE classification.

	Model 1 (All variables that were significant in univariate analysis)			Model 2 (Multivariate analysis by backward stepwise variable selection)			Model 3 (Multivariate analysis by forward stepwise variable selection)		
Variable	Odds Ratio (95% CI)	*p*-value	Score	Odds Ratio (95% CI)	*p*-value	Score	Odds Ratio (95% CI)	*p*-value	Score
**Baseline characteristics**									
** Body-mass index > 27.5 kg/m** ^**2**^	2.32 (1.43–3.75)	0.001	2	2.36 (1.24–4.46)	0.009	2	2.46 (1.32–4.55)	0.004	2
** Diabetes mellitus**	1.68 (1.06–2.67)	0.027	2	1.70 (0.91–3.15)	0.095	2			
** Alcoholic liver cirrhosis**	2.50 (1.42–4.37)	0.001	3						
** Serum albumin level < 3.5 mg/dl**	2.06 (1.37–3.10)	0.001	2	1.81 (1.07–3.06)	0.027	2	1.76 (1.05–2.94)	0.032	2
** MELD score > 20**	2.97 (1.96–4.50)	<0.001	3	1.94 (1.11–3.38)	0.019	2	2.01 (1.17–3.44)	0.011	2
** CTP score > 10**	2.72 (1.83–4.04)	<0.001	3						
** Child class C**	2.72 (1.83–4.04)	<0.001	3						
**Donor factors**									
** Estimated GRWR < 0.8**	2.52 (1.45–4.39)	<0.001	3	1.95 (0.89–4.30)	0.019	2			
**Operative details**									
** Operation time > 600 min**	1.83 (1.22–2.76)	0.004	2	1.65 (0.96–2.84)	0.070	2	1.81 (1.07–3.06)	0.027	2
** Cold ischemic time > 100 min**	1.67 (1.10–2.54)	0.015	2						
** Warm ischemic time > 40 min**	2.18 (1.46–3.26)	<0.001	2	2.68 (1.57–4.56)	<0.0011	3	2.61 (1.55–4.38)	<0.001	3
** Postreperfusion syndrome**	2.59 (1.59–4.20)	<0.001	3	3.12 (1.60–6.06)	0.001	3	2.96 (1.55–4.38)	0.001	3
** Norepinephrine infusion > 0.1 μg/kg/min**	1.80 (1.16–2.80)	0.009	2						
** Mean blood glucose during the day of surgery >150 mg/dl**	1.90 (1.29–2.78)	0.001	2	1.54 (0.93–2.56)	0.094	2	1.66 (1.01–2.70)	0.044	2
** pRBC transfusion > 5 units**	2.83 (1.68–4.78)	<0.001	3	0.47 (0.22–1.01)	0.052	1			
** FFP transfusion > 5 units**	2.70 (1.69–4.31)	<0.001	3						
** Platelet transfusion > 6 units**	5.80 (3.77–8.94)	<0.001	6	1.84 (0.92–3.68)	0.083	2			
** Cryoprecipitate transfusion > 6 units**	7.42 (4.85–11.36)	<0.001	7	3.51 (1.75–7.03)	<0.001	4	4.96 (2.84–8.64)	<0.001	5
** Blood loss per body weight > 60 ml/kg**	6.95 (4.52–10.69)	<0.001	7	5.00 (2.54–9.81)	<0.001	5	4.05 (2.28–7.21)	<0.001	4
**Postoperative factors**									
** CNI use without combined Mycophenolate mofetil**	2.06 (1.40–3.04)	<0.001	2	1.95 (1.17–3.26)	0.011	2	1.87 (1.14–3.06)	0.013	2

MELD score = model for end-stage liver disease score; CTP score = Child-Turcotte-Pugh score; GRWR = graft to recipient body weight ratio; GFR = glomerular filtration rate; pRBC = packed red blood cell; FFP = fresh frozen plasma; CNI = calcineurin inhibitor; CI = confidence interval.

**Fig 3:**
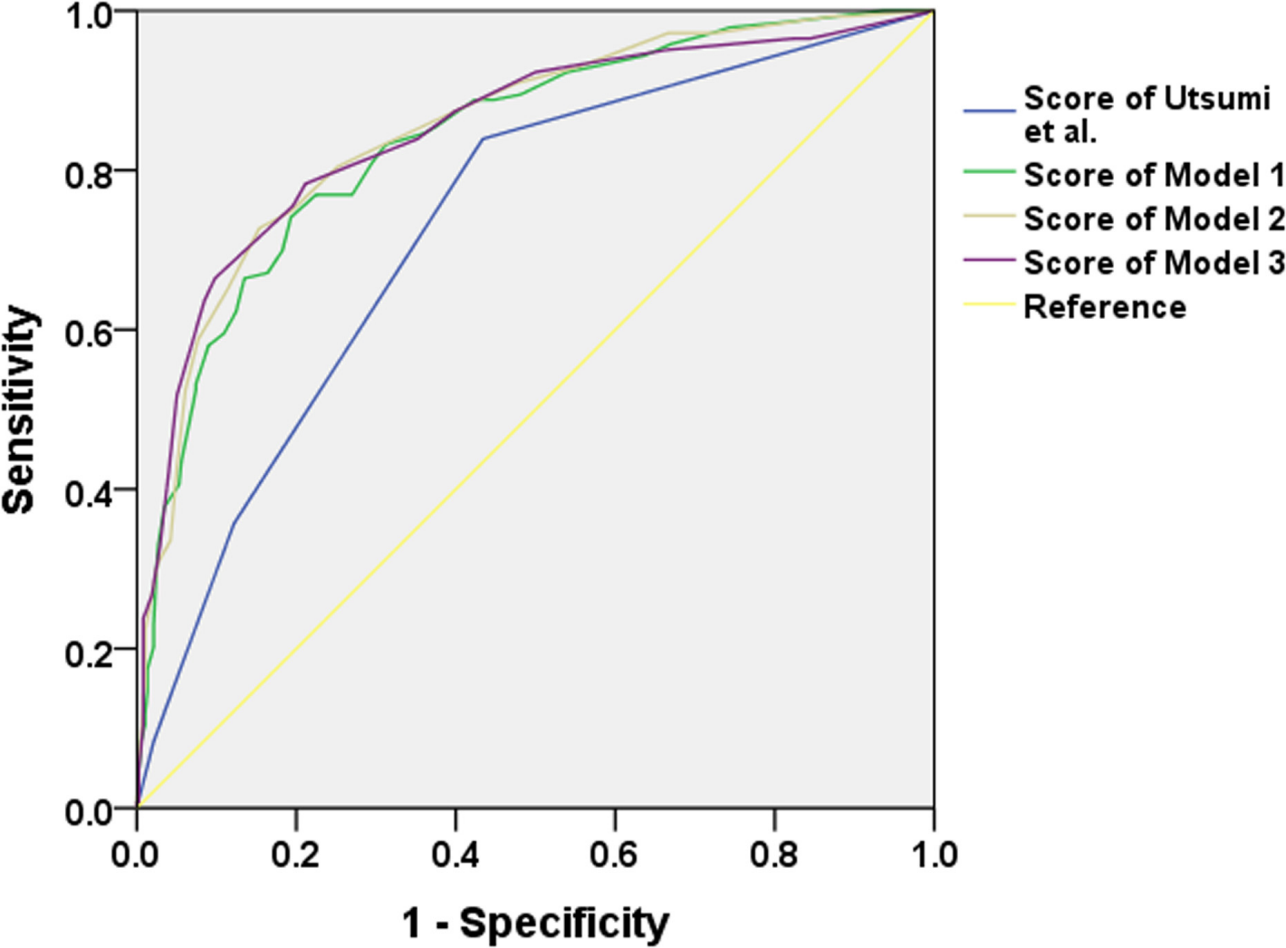
Receiver operating characteristic curves for prediction of AKI by the risk models of this study and previous risk score by Utsumi et al. Our risk models showed a significantly better performance in the comparison of AUC than the previous one.

All three risk models produced good discrimination and calibration when it was tested in our study cohort (Table 3). The AUCs of the risk model 1, 2, 3 were 0.85 (95% CI 0.81–0.89), 0.86 (95% CI 0.82–0.90), and 0.85 (95% CI 0.81–0.89). The risk models of the present study had better discriminative ability, with no overlapped 95% CI of that obtained by Utsumi et al. (Table 3)(Fig 3) [1]. According to Delong’s method, our three risk models showed significantly better performance in the comparison of AUC than the previous risk score (p<0.001, all). When we applied our risk models to randomly generated validation samples by way of cross-validation, our risk models retained good discriminative power (Table 3).

**Table 3:** Comparison between the risk models of the present and previous study.

	Study cohort (n = 538)	Cross-validation^a^	Hosmer-Lemeshow Goodness of fit			Comparison of AUC
Risk model	AUC (95% CI)	AUC (95% CI)	*χ* ^2^ statistic	*df*	*p*-value	p-value^b^
**Risk model 1**	0.85 (0.81–0.89)	0.85 (0.81–0.88)	6.32	8	0.611	<0.001
**Risk model 2**	0.86 (0.82–0.90)	0.86 (0.82–0.89)	2.17	8	0.975	<0.001
**Risk model 3**	0.85 (0.81–0.89)	0.86 (0.82–0.90)	12.34	8	0.137	<0.001
**Utsumi et al.**	0.73 (0.68–0.78)	0.72 (0.67–0.79)	3.79	5	0.580	

AUC = area under the receiver operating characteristic curve; CI = confidence interval.

^a^ The performance of each risk scoring model was evaluated by 10-fold cross-validation with an equal number of mutually exclusive ten subgroups of our cohort.

^b^ P-values were obtained by DeLong’s method of comparing AUC in study cohort between a given model and the reference model (reference model: risk model of Utsumi et al.).

## Discussion

In this retrospective observational study, we determined the incidence and risk factors for AKI after the living donor liver transplantation using the RIFLE criteria focusing on the potentially modifiable risk factors with relatively large number of LDLT cases. Among the independent variables revealed by multivariate logistic regression analysis, low serum albumin, postreperfusion syndrome, intraoperative hyperglycemia and CNI use without combined MMF use were notable as potentially modifiable risk factors. Preoperative uric acid level and vasopressor use were not included in the independent risk factors. We developed three clinical risk models to predict AKI after LDLT and validated them in the randomly selected subgroups of our cohort. Our risk models performed better in the comparison of AUC than a previously reported score for LDLT [1].

We found a relatively lower incidence of post-transplant AKI than was found in previous studies, which report incidences from 20.4% to 64.1% [1–3, 7–9, 12, 13, 15]. Previous studies suggested that the incidence of AKI after LDLT is between 29.0% and 63.1% [1, 8, 9, 13, 14]. This large discrepancy is possibly due to variable AKI definitions and/or inconsistencies in the postoperative management during which renal dysfunction was identified. Utsumi et al. [1] reported that the incidence of AKI (defined by RIFLE criteria) within 28 days of LDLT is 60.5%, which is much higher than that identified in our study. This difference may be due to different baseline liver disease type, baseline renal function, surgical technique, GRWR, and perioperative care. In our study population, hepatocellular carcinoma (HCC) accounted for 60.3% of cases and hepatitis B virus was the predominant cause of HCC. Despite the different AKI incidences, the risk factors of post-transplantation AKI in our study were similar to the previous study, although we investigated several more potential risk factors. The in-hospital mortality and one-year mortality of the AKI patients were 17.7% and 19.7%, which were similar to or slightly lower than those in other studies [1, 2].

CNI use is an established cause of renal dysfunction [37, 38]. We confirmed that CNI dose reduction with concurrent MMF use was associated with decreased AKI incidence, and CNI use without combined MMF was an independent risk factor of AKI after matching. Patients with high CNI trough levels or any related side effects were treated with MMF plus reduced-dose CNI in our institution during the study period. Two previous studies found that, although the combined use of MMF and CNI appeared to protect renal function as compared to using CNIs alone, there was no statistically significant relationship between MMF and renal function [1, 9]. Instead, overexposure to CNI was associated with postoperative AKI [1, 39]. This may be because we used MMF with reducing CNI dose in cases of high CNI trough levels or any presumed CNI toxicities, while Utsumi et al.[1] used MMF as the initial immunosuppression, or added it in cases of CNI toxicity. In line with this, previous studies have demonstrated that MMF introduction with CNI minimization resulted in a lower postoperative serum creatinine and improved GFR [40–42].

Postreperfusion syndrome was identified as an independent risk factor for AKI in this study even after matching. Previous studies have reported that postreperfusion syndrome was associated with adverse postoperative outcome including length of hospital stay and lower early survival [22, 43]. However, these studies reported different results regarding the effects of postreperfusion syndrome on postoperative renal function. Another previous large retrospective analysis of postreperfusion syndrome revealed that hemodynamic recovery after postreperfusion syndrome can be delayed until hepatic artery reperfusion [44]. This long period of hypotension can impair renal function and ischemia/reperfusion injury produced by organ reperfusion can induce a systemic inflammatory response that may deteriorate renal function [45]. Previous randomized trials showed that pharmacologic pretreatment including epinephrine and phenylephrine or serine protease inhibitor before reperfusion of the liver graft significantly reduced the incidence of postreperfusion syndrome [28, 46]. Therefore, prospective trials with a sufficient power are required to test the hypothesis that these pretreatments can also decrease the incidence of postoperative AKI after LDLT.

Preoperative hypoalbuminemia has been identified as a risk factor of post-LT AKI in this study, which is consistent with previous studies [15, 47]. Serum albumin itself has been reported to have a renoprotective effect by improving renal perfusion, inhibiting apoptosis of renal tubular cells, and promoting the proliferation of renal tubular cells [48–50]. However, low serum albumin level is a marker for overall sickness or pre-existing renal dysfunction, which may not be modifiable in a short time, and it is questionable whether artificial augmentation of albumin would decrease the incidence of postoperative AKI. An adequately powered randomized controlled study is required to confirm preoperative and/or pre- or intraoperative correction of hypoalbuminemia can really reduce the postoperative AKI incidence after LDLT. There has been no large randomized controlled trial which tested this hypothesis in patients undergoing LDLT, although a recent multi-center randomized trial disputed this hypothesis in patients with severe sepsis or septic shock [51].

Hyperglycemia during the day of surgery was associated with postoperative AKI in univariate analysis and hyperglycemia >150 mg/dl was an independent predictor in multivariate analysis. Previous studies reported that tight blood glucose control (<110 or <150 mg/dl) was associated with reduction in AKI and survival rate in cardiac surgical patients and critically ill patients [52–54]. Although a recent large randomized controlled trial reported that conventional glucose control target of 180 mg/dl or less resulted in lower mortality than intensive control target of 81 to 108 mg/dl [55], a meta-analysis including the NICE-SUGAR trial concluded intensive glucose control may be beneficial to surgical ICU patients [56]. In transplantation, several studies reported that hyperglycemia may increase the rate of postoperative infection and impair wound healing [24, 57]. However, the effect of perioperative hyperglycemia on the AKI after LT has not been fully evaluated and the appropriate glucose control target is still controversial. We chose 150 and 180 mg/dl as cutoffs according to the previous studies [57, 58], and there was no patients whose mean glucose was below 108 mg/dl.

Many of the other AKI predictors that we identified were similar to those previously recognized. Obesity and history of diabetes mellitus were also significant risk factor in previous studies [1, 59]. MELD score has been frequently reported to be a risk factor [2, 7, 8, 12]. Our results supported previous findings that significant intraoperative blood loss [1, 13] and large transfusion amount [3, 8, 15] are also risk factor for post-LT AKI. The use of hydroxyethyl starch was associated with poor renal function in kidney-transplant recipient or in severe sepsis patients [60, 61]. Although the amount of colloid used during operation was examined in this study, it was not associated with postoperative AKI. A small GRWR was associated with post-LT AKI in this study, which was consistent with previous studies [1, 62].

### Limitations

This study has limitations. First, this study only establishes association but not causality due to the retrospective observational study design. Prospective randomized trials are required to demonstrate whether modification of independent risk factors can really reduce the incidence of AKI after LDLT. Second, external validity of this study is limited because the data used to derive the risk score were obtained from only a single center. The risk score should therefore be validated prospectively at other centers to demonstrate its applicability. Third, although we examined the AKI incidences during postoperative one month, the possible post-transplant complications during this period which might be related to AKI have not been fully investigated. A previous study has reported that predictors for late postoperative acute renal failure (ARF) differ from early ARF and correspond to postoperative parameters including infection and surgical reoperation [47].

### Conclusion

Our risk models for post-LT AKI would provide accurate prediction and risk stratification about the risk of postoperative AKI in patients undergoing LDLT. In addition to unmodifiable previously-known risk factors including high MELD score, long surgery time, excessive blood loss, the present study identified low serum albumin, postreperfusion syndrome, hyperglycemia, and postoperative CNI use without combined MMF as potentially modifiable risk factors for AKI after LDLT. Prospective randomized trials are required to address whether artificial modification of these risk factors would decrease postoperative AKI in LDLT. CNI dose should be minimized with concomitant MMF introduction.

## Supporting Information

S1 File A dataset for the present study. (XLSX)
